# Different types of cell death and their shift in shaping disease

**DOI:** 10.1038/s41420-023-01581-0

**Published:** 2023-08-04

**Authors:** Sikou Shen, Yina Shao, Chenghua Li

**Affiliations:** 1 State Key Laboratory for Managing Biotic and Chemical Threats to the Quality and Safety of Agro-products, Ningbo University, Ningbo, 315211 PR China; 2 Laboratory for Marine Fisheries Science and Food Production Processes, Qingdao National Laboratory for Marine Science and Technology, Qingdao, 266071 PR China

**Keywords:** Apoptosis, Macroautophagy

## Abstract

Cell death is the irreversible stop of life. It is also the basic physiological process of all organisms which involved in the embryonic development, organ maintenance and autoimmunity of the body. In recent years, we have gained more comprehension of the mechanism in cell death and have basically clarified the different types of "programmed cell death", such as apoptosis, necroptosis, autophagy, and pyroptosis, and identified some key genes in these processes. However, in these previous studies, the conversion between different cell death modes and their application in diseases are rarely explored. To sum up, although many valued discoveries have been discovered in the field of cell death in recent years, there are still many unknown problems to be solved in this field. Facts have proved that cell death is a very complex game, and a series of core players have the ability to destroy the delicate balance of the cell environment, from survival to death, from anti-inflammatory to pro-inflammatory. With the thorough research of the complex regulatory mechanism of cell death, there will certainly be exciting new research in this field in the next few years. The sake of this paper is to emphasize the complex mechanism of overturning the balance between different cell fates and provide relevant theoretical basis for the connection between cell death transformation and disease treatment in the future.

## Facts

- Programmed cell death (PCD) is necessary for the normal development and homeostasis maintenance of organisms, and it is closely regulated by signals.
- At present, the mechanisms of different programmed cell death have been studied relatively clearly, such as apoptosis, pyroptosis, autophagy, etc.
- The mechanisms of different programmed cell death (PCD) are closely related to the pathogenesis of various diseases, and some have progressed to clinical trials, identifying drug formulations that are key participants in the cell death signaling pathway.
- At present, it has been proven that different programmed cell death modes can be transformed into each other through different gene expressions, providing new ideas for the role of different forms of cell death in related diseases and searching for potential drug targets.

## Questions

- Is there really a transition in the occurrence and development of programmed cell death with different order of occurrence in various diseases?
- Can the conversion between different programmed cell death by regulating the expression of key genes truly alleviate the occurrence and development of diseases?
- How to achieve a subtle balance between different cell death modes by regulating the expression of different genes during the occurrence and development of diseases to achieve the best therapeutic effect?

## Introduction

Cell death occurs when cells are unable to maintain foundational life functions. Traditionally, cell death is classified into accidental cell death (ACD) or regulated cell death (RCD) [1]. The ACD is an uncontrolled biological process, whereas RCD involves a signaling cascade in which effector molecules participate [1]. Among them, RCD is known as programmed cell death (PCD) also, which occurs under physiological terms. Depending on its morphological appearances, enzymatic criteria functions or immunological features, different ways of cell death can be sorted [2] (Table 1). So far, the researches focus on apoptosis, pyroptosis, necroptosis and autophagy mainly, which all belong to programmed cell death (PCD).

**Table 1:** Cell death modes and its characteristics.

	Apoptosis	Autophagy	Pyroptosis	Necroptosis	Necrosis
Properties	PCD	PCD	PCD	PCD	RCD
Cell morphology	Cells shrink	Produces vacuoles	Cell swelling and deformation,	Cell swelling and deformation,	Cell swelling and deformation
Cell membrane	Cell membrane structure is complete	Cell membrane structure is complete	Cell membrane rupture	cell membrane rupture,	Cell membrane rupture
Cell organelles	Organelles are complete	Cells are swallowed by autophagosomes and dissolved by lysosome	Organelle deformation	Organelle deformation or swelling	Organelle deformation or swelling
DNA	degrade to 180–200 bp and its integer	Random degradation	Random degradation	Random degradation	Random degradation

When organisms receive physiological or pathological signals, cells will respond accordingly—that is, produce various cell deaths to maintain the normal function of the living body as much as possible. Generally, autophagy usually precedes apoptosis and then starts apoptosis [3]. When the apoptotic cells are not cleared in time, it will lead to programmed cell necrosis or cell necrosis, that is, autophagy has the highest survival advantage [3, 4]. The second is apoptosis and programmed cell necrosis, and the survival advantage of cell necrosis is the lowest. For a long time, cell death pathways have been considered non overlapping completely, but runs in parallel [5]. However, recent advances showed, we have begun to recognize that these cell death processes modes can interact with each other through the interconnected, or even overlapped signaling pathways, and the ultimate cell fortune is the result of the interaction of different cell death modes, which is related closely with the development of diseases [6]. They interact with each other and jointly regulate the occurrence and outcome of the disease. These multiple cell death modes, such as autophagy, ferroptosis, pyroptosis, apoptosis and necroptosis, show a synergistic anti-tumor immune response and may inhibit anti-tumor immune response, which can help treat tumor cells without producing drug resistance [7]. Therefore, to explore the multi-layered relevance among anti-tumor immunity, non-apoptotic RCD and the potential targeted application of non-apoptotic RCD, combined with immunotherapy are able to play a strong anti-tumor activity [8].

More research is needed to determine the interaction among different signal pathways in cell death, as well as the unique effector molecules of each cell type. To summarize, it is urgent to explore the molecular regulatory mechanism of cell death in different diseases, which has great important scientific significance for deepening the basic research on the immunity of organism, revealing the pathogenesis of diseases and to prevent the occurrence and development of diseases. The purpose of this paper is to summarize the transformation among several classical cell death modes in mammals, so as to enlighten the character of cell death in the pathogenesis of various diseases, providing new ideas and promoting new treatment targets for people to find and study disease treatment.

## Apoptosis

**Fig. 1:**
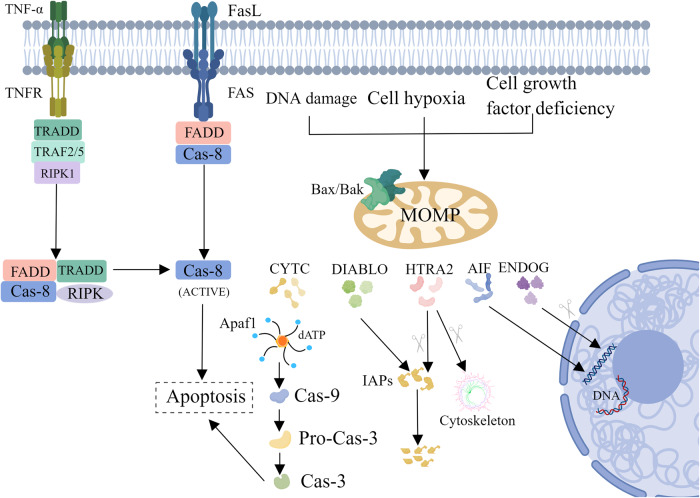
Two classical signaling pathways for apoptosis: the exogenous apoptotic signaling pathway and the endogenous apoptotic signaling pathway. Exposure to cellular insults, such as DNA damage, oxidative stress, and lack of cell growth factors, resulting the oligomerization of the BAX/BAK and heterotopic to the mitochondrial membrane, causing the permeability conversion of the mitochondrial membrane, releasing CYTC (Cytochrome C). Combined with Caspase-9, APAF1 (Apoptotic Peptidase Activating Factor 1), etc. to form an apoptosis complex that activates Caspase-9, then activates the downstream Caspase-3/7, and Caspase-3/7 will cut the downstream substrate to cause intrinsic apoptosis. Exposure to TNF- α, FASL, the downstream Caspase-8 will be activated, and the activated Caspase-8 will activate cascade reactions of caspases to inducing extrinsic apoptosis.

Apoptosis is mainly mediated by caspases protein family, which can be divided into endogenous apoptosis and exogenous apoptosis (Fig. 1). Cells that die in the form of apoptosis will exhibit membrane shrinkage, chromatin agglutination, apoptotic bodies formation, cytoskeleton disintegration, etc., of which the change of the nucleus is the most significant [9]. As we all known, apoptosis is a basic biological phenomenon of cell, which plays an important role in removing of superfluous, irreversibly damaged, or potentially harmful cells [10]. In contrast to pyroptosis and necroptosis, due to the presence of intact cell membranes in early stage of apoptosis, apoptosis is judged to be immunological silence, even anti-inflammatory [11, 12]. The comprehensive and irreversible mitochondrial outer membrane permeabilization (MOMP) is the key procedure of intrinsic apoptosis [13, 14]. When receive a stimulus(DNA damage、growth factors), Bad/Bax proteins, which belong to apoptosis promoting protein family will form oligomer complex, then transfer from the cytoplasm onto the outer mitochondrial membrane, thus change the permeabilization of outer membrane and transmembrane potential to release apoptosis correlated factors [14–16]. Factors released into cytoplasm, apoptotic protease activating factor-1(Apaf-1), cytochrome c and dATP, will compose as apoptotic bodies, subsequently activate caspase-9. In the end, caspase-3/7(executioner caspases) will be catalyzed by caspase-9 to carry out proteolytic activation to induce apoptosis [17, 18]. In addition to the cytochrome C-mediated apoptosis mentioned above, there is another apoptosis pathway in the mitochondrial pathway that does not rely on classical caspases, which is mediated by the apoptotic inducible factor (AIF) protein [19]. AIF is present inside the mitochondria usually. When cells are exposed to internal apoptosis-stimulating factors, AIF can be transfer the site from the mitochondria into the cytoplasm and ultimately causing DNA damage in the nucleus and leading to cell death [20]. The critical protein of the exogenous apoptosis pathway are two patterns of plasma membrane receptors: dependence receptors and death receptors [21–23]. The common character of death receptor is the possession of an extracellular domains full of Cysteine and an intracellular death domain [24]. There are a series of typical death receptors: DR3/4/5, Fas, TNFR1/2 and so on [25]. When death receptors bind to their ligands in cell surface, they form stable trimer complexes which is capable of recruiting adapter proteins(caspase-8), composing the Death Induction Signaling Complex (DISC) belongs to a signaling complex [26–28]. The DISC plays a homologous role like the apoptotic bodies in mitoptosis that is capable to cleave and activate the effect apoptosis protease(caspase-3) [29]. The activated caspase-3 induce a cascade of reactions later that trigger the apoptosis, which eventually leads to cell apoptosis [30].

## Pyroptosis

**Fig. 2:**
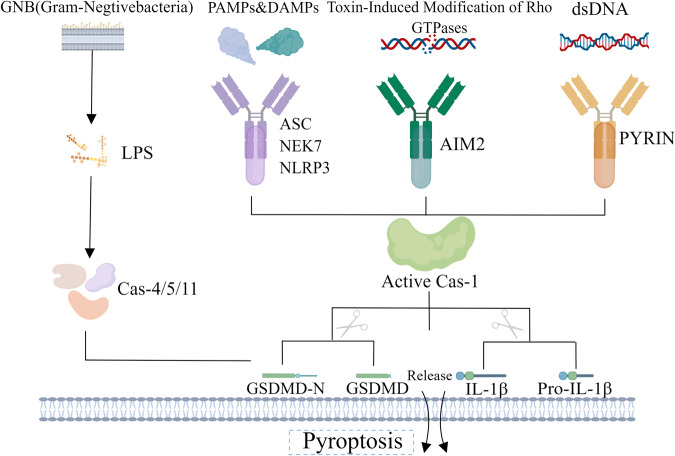
The signal pathway of cell pyroptosis: the cas-1 independent of pyroptosis and the cas-1 dependent of pyroptosis. Under the stimulation of bacteria, viruses and other signals, the PRRs (pattern recognition receptors) in the cell recognize these signals, and through the Apoptosis-associated speck-like protein (ASC) and the precursor of Caspase-1, forms a multi protein complex to activate Caspase-1. The activated Caspase-1 cuts Gasdermin D, forms a peptide segment containing the nitrogen terminal active domain of Gasdermin D, induces cell membrane perforation, cell rupture, releases content, and causes inflammatory reaction; On the other hand, activated Caspase-1 cuts the precursor of IL-18 and IL-1β to generate active IL-1β and IL-18, releasing into the extracellular space, recruiting inflammatory cells to aggregate and amplify the inflammatory response. Under the stimulation of signals such as bacteria, Caspase-4, 5, and 11 are activated, and the activated Caspase-4, 5, and 11 can cleave Gasdermin D, IL-18 as well as IL-1 β to inducing pryroptosis and inflammatory reaction.

There are classical and non-classical pathways in pyroptosis [31], the classic pathway is usually done in two parts (Fig. 2). In the first step-‘initiate signal’, a majority of proteins are induced to express by activating NF-kB pathway, these kinds of proteins will be a part of inflammatory bodies [32] inflammatory bodies own a cytoplasmic pattern recognition receptor (PRR), an adapter protein and pro-caspase-1 [33]. Subsequently, at the second procedure, caspase-1 will cleave he N-terminal fragments of gasdermin D that it can locate in plasm membrane and form pores, leading to pyroptosis. In the meantime, the hydrolysis and activation of pro-IL-1β and pro-IL-18 will generate Its pro-inflammatory forms-IL1β and IL18, eventually eliciting inflammation and immune responses [34–36]. Other than that, pyroptosis can be accomplished by a non-classical path of LPS, in which the caspase-4\5\11 [mice: caspase-11; human: caspase4\5] are activated to cleave GSDMD to induce pyroptosis [37–39].

## Necroptosis

**Fig. 3:**
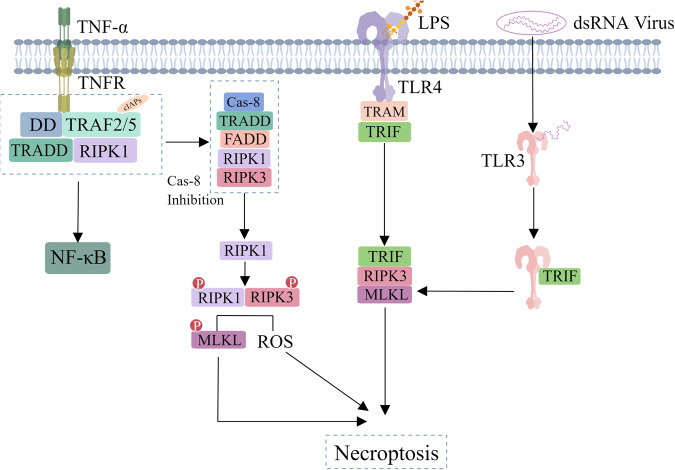
Classical signal pathways of cell necroptosis, which are induced by a variety of innate immune signal transduction including the stimulation of ds DNA virus, and toll-like receptor and death receptor signals. At TNF- α Under stimulation, while inhibiting Caspase-8 in cells, RIP1 in the apoptotic complex dissociates and self phosphorylates with RIP3, causing oligomerization and phosphorylation of downstream MLKL or an increase in ROS, ultimately leading to necrotic apoptosis.

It has long been thought that the caspases protein family is closely related to apoptosis. However, studies have found that inhibiting caspases does not prevent cell death caused by death receptors, and this death has certain necrotic characteristics [40] (Fig. 3). In 2005, studies clarified this new mode of cell death and defined it as necroptosis. Necrosis and necroptosis share similar morphological characteristics but dies in a regulated, independent-caspases programmed cell death [41]. Under the stimulation of TNF-α, the TNFR1 will combine with TNF-α, and recruit a series of proteins to form complex Ι, if caspase-8 is inhibited, RIPK1 in complex I will recruit RIPK3 by their respective RIP homotypic interaction motif (RHIM) domains, and compose necrosome by self- phosphorylation [42–44]. Necrosome will further recruit MLKL that phosphorylated by RIPK3, then phosphorylated MLKL shift from monomerization to oligomerization to combines inositol phosphate and cardiac phospholipids. Finally, the necrosome transfer from cytoplasm to cell membrane or organelle membrane to form permeable pores, destroy integrity of membrane, thus result in necroptosis [41, 45–47].

## Autophagy

**Fig. 4:**
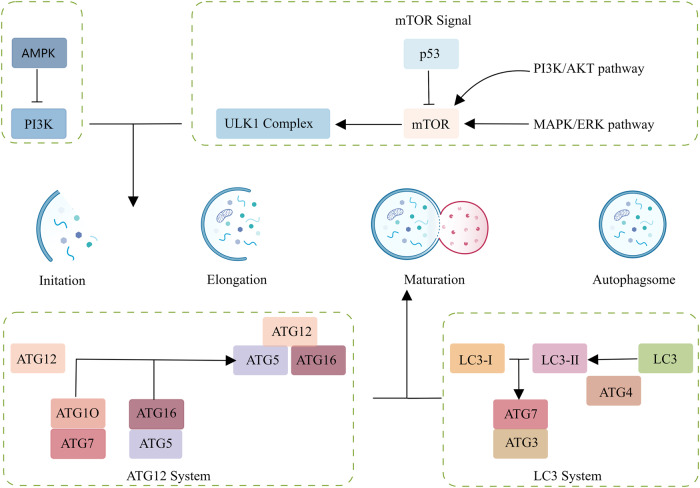
The five processes of autophagy and their roles in autophagy regulation. The ART/MAPK pathway activates the mTOR of cells, thereby inhibiting autophagy. The AMPK/P53 pathway inhibits cell mTOR and promotes autophagy. ULK1 complex is a key complex formed by connecting upstream mTOR and downstream autophagosomes. When cells are in a state of malnutrition and hunger, mTOR is inactivated, catalyzing the phosphorylation of ULK1 and promoting autophagy. When cells are in a healthy state, mTOR is activated and binds to ULK1, inhibiting ULK1 phosphorylation and inhibiting autophagy. The maturation of Autophagosome requires the participation of ATG and LC3. Atg12 is coupled to Atg5 through the ubiquitin reaction of Atg7 and Atg10, and then linked to Atg16 through non covalent reaction to form a larger complex to promote the generation of Autophagosome. LC3, on the other hand, is cleaved by ATG4 hydrolase to generate cytoplasmic LC3-I, which is then connected to PE through the ubiquitin like reaction of ATG7 and ATG3 to generate LC3-II, which is then adsorbed onto the autophagosome membrane. The presence of LC3 in autophagosome and its conversion to LC3-II are used as indicators of autophagy.

The main function of autophagy is to achieve the metabolic needs of cells themselves and to update some organelles. Cells achieve this goal by engulfing their own cytoplasmic proteins or organelles and coating them into vesicles, then fusing with lysosomes to form autophagic lysosomes to degrade their contents [48]. Autophagy is divided into five stages: [49] autophagy induction phase, autophagic membrane vesicle nucleation phase, autophagosome formation phase and mature degradation phase (Fig. 4). Five signaling pathways take part in the autophagy. The PI3K-AkT pathway and the MAPK-ERK pathway will activate the mTOR, thus resulting in the inhibition of autophagy. On the contrast, the AMPK and P53 pathways suppress usually the mTOR, inducing autophagy. Among these contents, the ULK1 complex is the most important one, which serve as a bridge to connect upstream nutrient receptors(mTOR) with downstream autophagosome [50]. As for the ATG families, Atg12-Atg5 complex and LC3 complex can control the formation of autophagosome [51]. Atg12 is coupled to Atg5 through the ubiquitin reaction of Atg7 and Atg10, and then linked to Atg16 through non covalent reaction to form a larger complex to promote the generation of Autophagosome [52]. LC3, on the other hand, is cleaved by ATG4 hydrolase to generate cytoplasmic LC3-I, which is then connected to PE through the ubiquitin like reaction of ATG7 and ATG3 to generate LC3-II, which is then adsorbed onto the autophagosome membrane [53]. The presence of LC3 in autophagosome and its conversion to LC3-II are used as indicators of autophagy. The mammalian rapamycin target protein (mTOR) that is a receptor for a variety of amino acids and ATP is a key protein in the classical induction pathway of autophagy [54–56]. In normal cells, mTOR is activated by phosphorylation of certain cytokines, and autophagy remains at the basal level, but under stress conditions, mTOR activity will decrease, so the autophagy signaling pathway is activated, and autophagy is induced [57–60]. Autophagy-related complexes are then formed to initiate the nucleation of membrane vesicles, at which point ATG proteins bind to the membrane and autophagosomes are formed. Finally, autophagosomes and lysosomes combine to form autophagic lysosomes, the contents of which are degraded by lysosomes, and the small molecule degradants are returned to the cytoplasm to participate in the biosynthesis and metabolism process [61, 62].

## Conversion of these PCDs and their shift in shaping disease

**Fig. 5:**
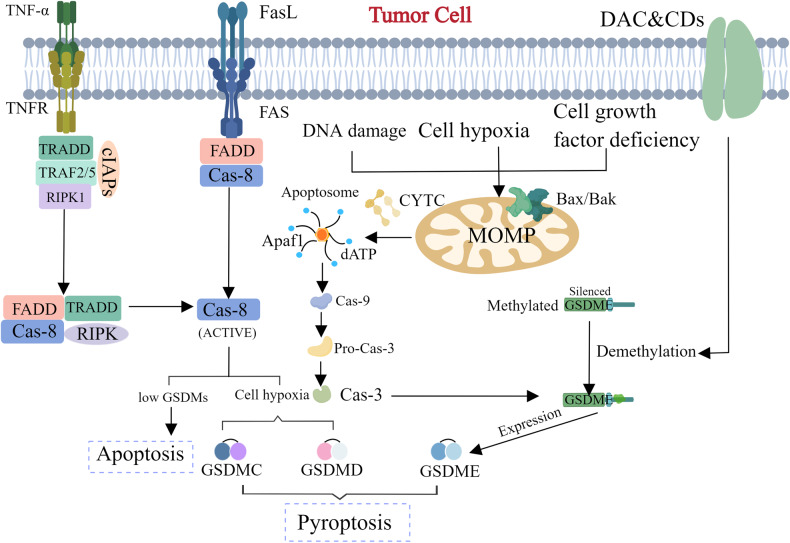
The transition from apoptosis to pyroptosis induced by GSDMs in tumor cells: the methylated GSDMs are silent, so tumor cells usually tend to apoptosis. With demethylated drugs, the cells can be led to pyroptosis, thus resisting the drug resistance caused by chemotherapy drugs and the reduction of apoptosis.

Although the mechanisms are considered differently, the connection between the four PCPs is widely recognized. For example, researches denotes that GSDME is the important intersection of apoptosis and pyroptosis, as a critical player in conversion of apoptosis and pyroptosis [63]. The GSDMs family of proteins which identified as a class of pore-forming proteins recently are the direct executioners of pyroptosis [64]. There are six species in humans, GSDM A to E and an additional DFNB59 [65]. With the exception of one additional member, the other proteins have two relatively conserved domains, GSDM NT and GSDM CT [66, 67]. The lipophilic nature of NTs allows them to bind to and punch holes in the plasma membrane, eventually causing the cells to penetrate and swell until the plasma membrane ruptures [32, 68]. Recent studies have shown that exist a kind of pyroptosis caused by GSDME and caspase-3 [69]. In this case, caspase-3 specifically cleaved GSDME in linker domain to generate N-terminal fragments of GSDME, thus punch holes in plasm membranes to induce pyroptosis instead of apoptosis [70]. Moreover, this switch depends on the expression level of GSDME, when GSDME highly expressed, resulted in pyroptosis, when expression is low, convert to apoptosis [71]. In other words, GSDME can switch apoptosis induced by TNF-α or chemotherapy drugs rely on capase-3 to pyroptosis. GSDME is not expressed in most cancer cells, but is expressed in a variety of normal tissues [72]. Most cells in humans exhibit dependent-caspase-3 cleavage-induced pyroptosis under stimulation by chemotherapy drugs. Mice that do not express GSDME are spared a variety of tissue damage and weight loss caused by chemotherapy drugs [73]. All of this evidence suggests an unexpected conclusion that Cas-3 activation can activate pyroptosis by cutting GSDME, which opens up a new picture for chemotherapy drug treatment of cancer [71] (Fig. 5).

The transition between apoptosis and necroptosis depends on RIPK1 and RIPK3 [74]. Studies have shown that receptor-interacting proteins (RIP) are important intersections of cell survival and death, and play a key role in apoptosis and cell survival, necroptosis, etc. [75]. Studies have shown that a lack of caspase-8 during mouse embryonic development can lead to the termination of embryonic development, but this death can be recovered by knocking out the RIP3 or MLKL [76]. RIPs are a class of proteins with specific serine (Serine) and threonine (Threonine) kinase activity [77]. Which can be signaling molecules that regulate cell death or survival, participate in cellular stress responses, and initiate and regulate cellular stress responses for different stress signals such as inflammation, pathogenic infection, cell differentiation, and injury, and then determine the different survival directions of cells such as apoptosis, necrosis or survival. The RIP family consists of seven members, of which RIP1 and RIP3 are the most important regulatory proteins, and RIP1 is the intersection that determines cell survival and death [78]; RIP3 is the converter that determines how cells die [79]. The N-terminus of RIP1 contains a kinase domain (KD), and the C-terminus contains a death domain (DD) and a domain called RIP homotypic interaction motif (RHIM). RIP1 can both promote cell survival or induce cell death in cells by activating the NF-κB signaling pathway [80, 81]. RIP3 contains a KD domain at the N-side, and its C-side also contains an RHIM domain. RIP3 as a molecular switch of the cell death mode, its protein level is the key to controlling the survival or death of cells, if the expression of RIP3 is high, the cell will go to necrosis; RIP3 expression is low, and cells are moving toward apoptosis [82].

**Fig. 6:**
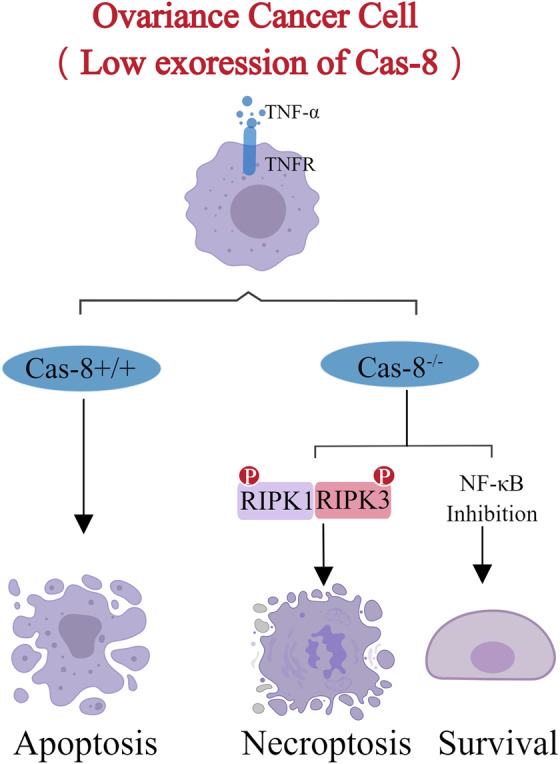
The transition between apoptosis and necroptosis in ovarian cancer cells: with the low expression of cas-8, the cells would rather go to survival than apoptosis. RIP1 and RIP3 can form necrotic bodies together, making the cells go to necroptosis, resisting the drug resistance caused by chemotherapy drugs, and making up for the weakened cell apoptosis.

Research shows that although cancer cells with high expression of Caspase8 die due to apoptosis, the loss of Caspase8 can down-regulate NF- κ B signal transduction, strengthen the stability of RIPK1 to promote the cell to necroptosis, which is of great significance to improve the anti-cancer strategy of ovarian cancer patients with low expression of Caspase8 [83] (Fig. 6).

Recent findings suggest that dysregulation of this necroptosis pathway affects many cardiovascular diseases, including atherosclerosis, myocardial infarction, stroke and concomitant ischemia-reperfusion injury, myocarditis, and thrombosis [84]. It has also been linked to lung disease which includes acute respiratory distress syndrome and acute and chronic lung injury. In addition to the aforementioned cardiovascular diseases, dysregulation of the RIPK3-mediated necrotic pathway has been linked to several neurodegenerative diseases, such as amyotrophic lateral sclerosis (ALS), Parkinson’s disease and multiple sclerosis [85–87].

**Fig. 7:**
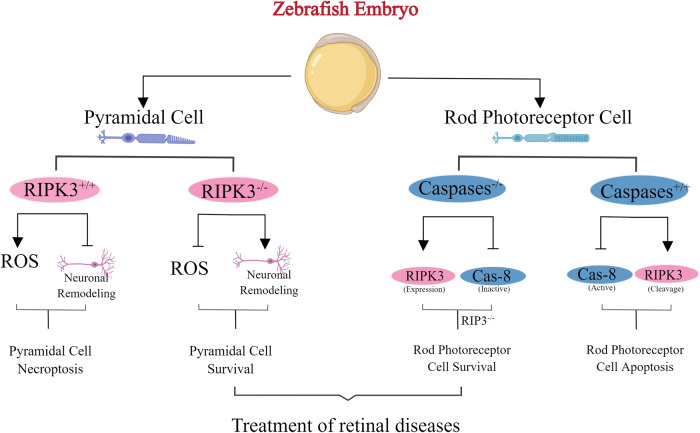
The conversion between apoptosis and necroptosis for the treatment of retinal diseases during zebrafish embryonic development: pyramidal cells will be reduced due to RIP3 dependent necroptosis, while rod photoreceptor cells will be reduced due to caspase-dependent apoptosis. Therefore, we can help treat retinal diseases by inhibiting different key genes in different types of cells and making them go to different ways.

In the rain frog-induced mouse model of acute pancreatitis, knockout of the RIP3 gene significantly reduced the necrosis of pancreatic cells, thereby contributing to the recovery of acute pancreatitis [88]. Deletion of RIP3 in mutant zebrafish embryos (pde6cw59) rescues dying cone photoreceptors by reducing reactive oxygen species formation and secondary neuronal remodeling in the retina. Furthermore, inhibiting RIP1 and RIP3 activity by pharmacologic delayed pyramidal cell death. In contrast, rod photoreceptor cell death produced through a caspase-dependent mechanism. Since cone and receptor cell death is a significant characteristics of many retinal diseases, switching methods for different cell death pathways may develop into an important general principle in treatment [89] (Fig. 7).

The study also found that there was a strong correlation between the aggravation of neonatal HI brain injury and the mode of cell death during hyperglycemia. Hyperglycemia will not enhance exogenous cell apoptosis, but will promote the increase of RIP1 and MLKL levels, while the cut of the executioner caspase and PARP1 will be reduced, making the cells turn to RIP1-dependent necroptosis [83].

In some circumstances, autophagy can inhibit apoptosis, which is one of the way for cells move towards to survive [90]. But sometimes, autophagy itself can also lead to cell death, or along with apoptosis, act as an auxiliary mechanism to induce cell death at the absence of apoptosis [91]. The two pathways are interrelated and mutually regulated. Studying and utilizing these interactions will help to further reveal the mechanism of occurrence and development of diseases such as tumors. Beclin1, an autophagy gene, is an important participant in the formation of autophagy and can also interact with apoptosis related cytokines [92]. On the one hand, Beclin1 can eliminate anti-apoptotic effect of Bcl-2 by combining its BH3 domain with anti-apoptotic proteins (Bcl-2, Bcl-xl and Bcl-w) [93]. On the other hand, BH3 can interact with some pro-apoptotic protein (Bak, Bax, Bad and Bok) to reduce autophagy [94]. In other words, the prominence regulatory role of Beclin1 in the process of autophagy is mainly realized through the interaction between BH3 domain and Bcl-2 family proteins [95, 96]. Other studies have shown that Beclin1 can not only bind to Bcl-2, but also interact with PI3KC3, and finally promote autophagy. In addition, Beclin1 can be used as a substrate of caspases and cleaved by caspases. After cleaved, Beclin1 loses autophagy function but promotes cell apoptosis [97].

**Fig. 8:**
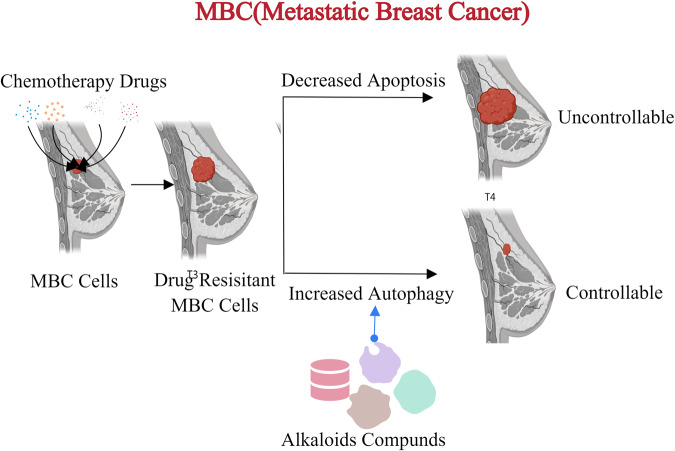
The conversion between apoptosis and autophagy for MBC. During the treatment of metastatic breast cancer, due to long-term chemotherapy, MBC cells will have a certain degree of drug resistance, which will reduce apoptosis and fail to achieve the therapeutic effect. At this time, some biotics can be used to stimulate the increase of autophagy and enhance the therapeutic effect.

Due to the development of chemotherapy resistance, metastatic breast cancer is basically determined to be an incurable disease [98]. Experiments have shown that resistance to chemotherapy drugs may be mainly caused by defects in cell apoptosis [99]. Studies have demonstrated that the apoptotic response of drug-resistant MCF-7 breast cancer cells to a series of drugs is significantly reduced, and the reduction of apoptosis in drug-resistant cells is balanced by the upregulation of autophagy, therefore, the efficacy of anti-cancer drugs can be enhanced through autophagy [100]. Currently, many new anticancer compounds have been extracted from natural products, such as these alkaloids, including liensinine, isoleucine, dauricine, etc [101]., which can stimulate AMPK-mTOR-dependent autophagy induction and autophagic cell death in anti-apoptotic cells (Fig. 8). Therefore, the treatment effect of anti-cancer drugs can be enhanced through autophagy mediated cell death mechanism [99].

**Fig. 9:**
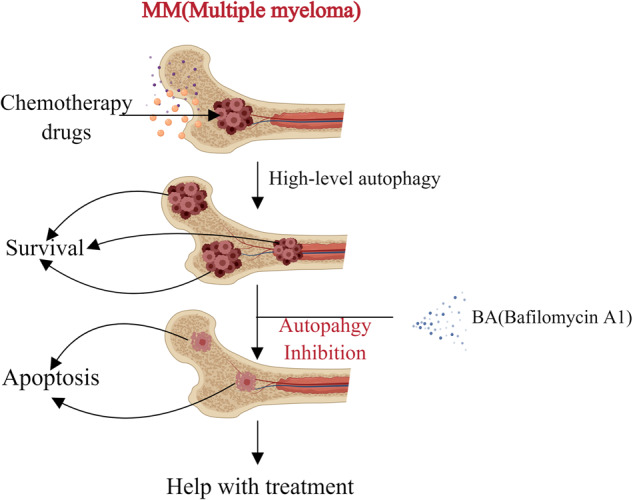
The conversion between apoptosis and autophagy for Multiple myeloma treatment: Multiple myeloma cells will produce high levels of autophagy during the treatment of chemotherapy drugs. A large number of cancer cells will move towards survival, greatly affecting the curative effect. BA can be used to inhibit autophagy, makes a part of cells move towards apoptosis, and help the treatment.

Multiple myeloma (MM) is a clonal plasma malignant tumor [102]. Due to the lack of effective treatment, the treatment effect of patients with confirmed MM is not optimistic. In fact, the level of autophagy in MM cells is significantly higher than that of normal cells [103]. It has been proved that inhibition of autophagy can cause MM cell death [104]. In addition, autophagy can protect MM cells from apoptosis under nutrient depletion conditions [105] and during drug resistance [106]. In other words, BA can inhibit autophagy of KM3 cells to increase BA-induced apoptosis of KM3 cells, thus helping to treatment (Fig. 9).

Some studies also showed that trehalose (Tre), a new autophagy activator, can inhibit caspase-dependent apoptosis pathway and promote autophagy recovery to reduce the cytotoxicity of cadmium to rPT cells [107]. Thus, cadmium-induced rPT cell death was significantly reduced.

There are also studies showing that overexpressed RIP3 can also phosphorylate GNAI3 and RGS19 through its kinase activity, thereby promoting autophagy to inhibit the growth of colon cancer cells HT29 [108]. That is to say, RIP3-regulated autophagy is related with the emergence and development of colon cancer, providing some potential application value for the clinical therapy of colon cancer.

## Significance of the conversion of PCDs for innate immunity and therapeutics

The Innate immune system can respond to different types of stress encountered by the body. According to different external stimuli, it can identify biomacromolecules with pathogen-associated molecular pattern (PAMP) or damage-associated molecular pattern (DAMP) through Pattern recognition receptor (PRR) to protect the host [109]. At the same time, pathogens have also come up with new strategies to avoid the treatment of the Innate immune system. As mentioned above (Figs. 6–9), many tumor cells silence genes related to PCDs, making it impossible for certain PCDs to occur, thereby exacerbating the condition. Moreover, during the embryonic development of Zebrafish, by clarifying the conversion between different cell death modes and inhibiting unwanted PCD, normal eye tissue development and body health can be ensured. In these cases, activating the conversion between PCDs through external means to enhance the death of diseased cells will be more conducive to combating pathogens and diseases.

In summary, the above results indicate that the activation of different PCDs is a common means for hosts to combat diseases. In a certain disease, the occurrence of PCDs is not singular, but overlapping [110]. Understanding the potential molecular mechanisms underlying the conversion between different PCDs has enabled us to identify more potential therapeutic targets and provide new ideas for disease control and treatment by clarifying molecular mechanisms for clinical drug testing and regulation.

## Conclusion

**Fig. 10:**
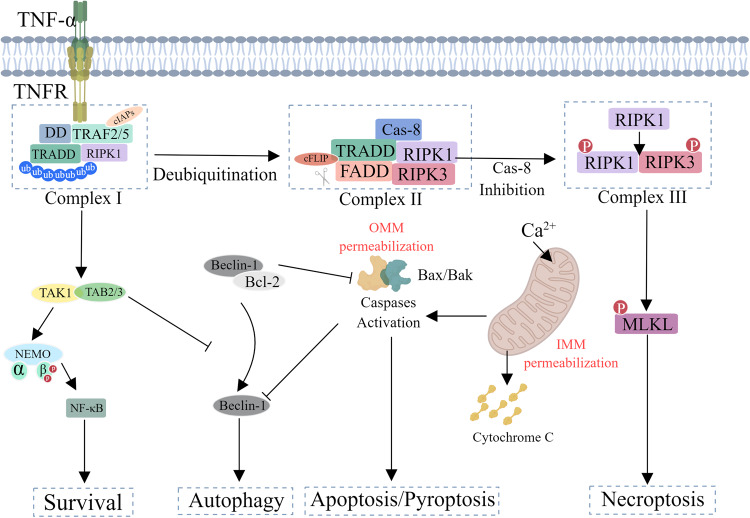
The specific conversion process of cell survival, autophagy, apoptosis, pyroptosis, and necroptosis under the stimulation of TNF-α. Cells are subjected to TNF- α After stimulation, TNFR recruits TRADD, TRAF2, RIP3, etc. to form complex 1, activating the NF where downstream TAK1, TAB2/3 are located- κ The B pathway promotes cell survival, and autophagy also occurs. When autophagy is excessive, autophagic cell death occurs. After de ubiquitination of RIPK1, RIPK3 and Caspase-8 are recruited to generate complex 2, which activates the cascade reaction of Cspases and causes cell apoptosis. When the lack of Vaspases-8 or Caspases-8 activity is inhibited, RIPK1 and RIPK3 will dissociate, forming complex 3, causing polymerization of MLKL.

**Fig. 11:**
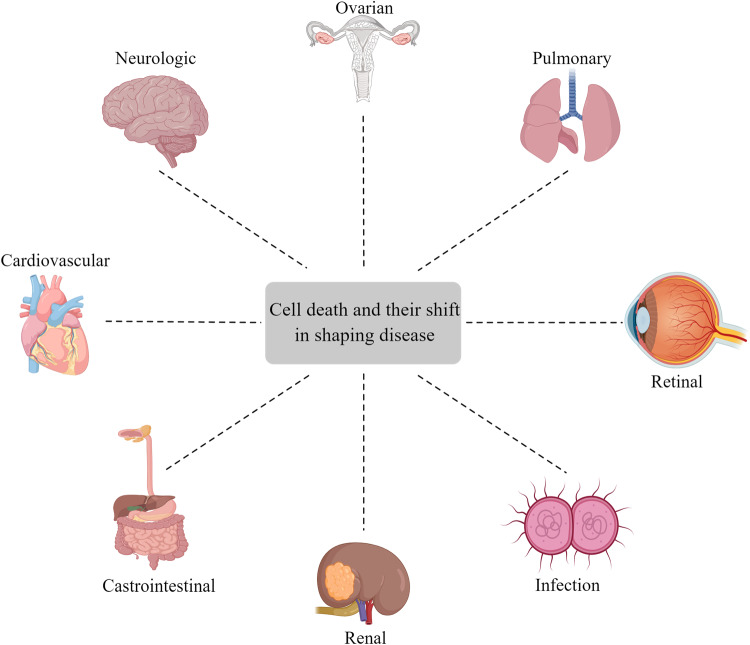
The connection and transformation between cell death modes are closely related to human diseases, especially in cancer and neurological diseases.

With the in-depth study and attention to the mechanism of cell death, researchers have found that different forms of cell death can be converted to each other and are closely related to the emergence and development of diseases (Fig. 10). Therefore, cell death has become an important entry point for the treatment of various cancers and infectious diseases at present (Fig. 11). Although we have conducted more in-depth research on the conversions between different cell death modes, it is still unknown how to achieve a subtle balance in the actual treatment of diseases to achieve the treatment of diseases and reduce the side effects of drugs. This greatly limits the development of the strategy from cell death to immune prevention. The occurrence of diseases is often the result of the interaction of multiple cell death modes. In the current environment of frequent occurrence of such diseases, starting from the molecular mechanism of cell death mode conversion, taking manual intervention and attempting to convert the cell death modes may be able to alleviate the side effects of some cell death modes to a certain extent. For example, when coral is infected by pathogens, coral cells will die in the form of necrosis with the activation of Caspase-3 and the cutting of GSDME, but this death can be recovered by inhibiting the cutting of GSDME by Caspase-3 [111].

In short, after seeing the good effect of the conversion between different cell death modes in the treatment of some mammalian diseases, we see the hope that the conversion from the molecular mechanism of cell death can further help the treatment of diseases and reduce the side effects of prognosis. To do this, we must try to switch the cell death mode by targeting key genes, avoid the regulation of abnormal cell death, achieve the subtle balance in the body, and achieve better therapeutic effect on the basis of exploring the molecular mechanism of different RCD in the body and finding the internal relationship between them.

## Data Availability

Data sharing not applicable to this article as no datasets were generated or analysed during the current study. All data generated or analyzed during this study are included in this published article or available from the corresponding author on reasonable request.
